# Bosentan and macitentan prevent the endothelial-to-mesenchymal transition (EndoMT) in systemic sclerosis: in vitro study

**DOI:** 10.1186/s13075-016-1122-y

**Published:** 2016-10-06

**Authors:** Claudio Corallo, Maurizio Cutolo, Bashar Kahaleh, Gianluca Pecetti, Antonio Montella, Chiara Chirico, Stefano Soldano, Ranuccio Nuti, Nicola Giordano

**Affiliations:** 1 Scleroderma Unit, Department of Medicine, Surgery and Neurosciences, University of Siena, 53100 Siena, Italy; 2 Research Laboratory and Academic Division of Clinical Rheumatology, Department of Internal Medicine, Institute for Research and Health Care (IRCCS), University of Genoa, San Martino, Genoa, Italy; 3 Division of Rheumatology and Immunology, College of Medicine, University of Toledo, Toledo, OH USA; 4 Medical and Scientific Direction, Actelion Pharmaceuticals Italy, Imola, Italy

**Keywords:** Systemic sclerosis, EndoMT, Microvascular endothelial cells, Bosentan, Macitentan

## Abstract

**Background:**

Systemic sclerosis (SSc) is characterized by early vascular abnormalities and subsequent fibroblast activation to myofibroblasts, leading to fibrosis. Recently, endothelial-to-mesenchymal transition (EndoMT), a complex biological process in which endothelial cells lose their specific markers and acquire a mesenchymal or myofibroblastic phenotype, has been reported in SSc. In the present study, we evaluated the ability of endothelin-1 (ET-1) dual receptor antagonists bosentan (BOS) and macitentan (MAC) to antagonize EndoMT in vitro.

**Methods:**

Ten women with limited SSc were enrolled. They underwent double skin biopsy (affected and nonaffected skin). Fibroblasts and microvascular endothelial cells (MVECs) were isolated from biopsies. We performed mono- or coculture of MVECs (isolated from nonaffected skin) with fibroblasts (isolated from affected skin and stimulated with ET-1 and transforming growth factor beta [TGF-β]). In cocultures, the MVEC layer was left undisturbed or was preincubated with BOS or MAC. After 48 h of coculture, MVECs were analyzed for their tube formation ability and for messenger RNA and protein expression of different vascular (CD31, vascular endothelial growth factor-A [VEGF-A], VEGF-A165b) and profibrotic (alpha-smooth muscle actin [α-SMA], collagen type I [Col I], TGF-β) molecules.

**Results:**

After 48 h, MVECs showed a reduced tube formation ability when cocultured with SSc fibroblasts. CD31 and VEGF-A resulted in downregulation, while VEGF-A165b, the antiangiogenic isoform, resulted in upregulation. At the same time, mesenchymal markers α-SMA, Col I, and TGF-β resulted in overexpression in MVECs. Tube formation ability was restored when MVECs were preincubated with BOS or MAC, also reducing the expression of mesenchymal markers and restoring CD31 expression and the imbalance between VEGF-A and VEGF-A165b.

**Conclusions:**

With this innovative EndoMT in vitro model realized by coculturing nonaffected MVECs with affected SSc fibroblasts, we show that the presence of a myofibroblast phenotype in the fibroblast layer, coupled with an ET-1-TGF-β synergic effect, is responsible for EndoMT. BOS and MAC seem able to antagonize this phenomenon in vitro, confirming previous evidence of endothelium-derived fibrosis in SSc and possible pharmacological interference.

## Background

Systemic sclerosis (SSc) is an autoimmune connective tissue disease characterized by cutaneous and visceral fibrosis coupled with widespread vascular pathology [1]. The first sign occurring in 90–98 % of patients with SSc is Raynaud’s phenomenon, an abnormal reactivity of digital microvasculature under cold and other stimuli [2, 3], which highlights the central role of microvascular damage in the pathogenesis of SSc [4]. In SSc, imbalance of endothelial signals, such as increased release of vasoconstrictory endothelin 1 (ET-1), thromboxane, and thrombomodulin, and reduction of prostaglandin I_2_ and nitric oxide are signs of the endothelial injury [5]. Although different molecules have been identified as key effectors of vascular remodeling in SSc, such as ET-1 and transforming growth factor beta (TGF-β), the mechanisms underlying SSc vasculopathy remain poorly understood [6]. There are two important issues: how the vascular damage can lead to the fibrotic process and the relationship between vessel disappearance and myofibroblast activation [7]. In fact, myofibroblasts in fibrotic tissues are derived from at least three sources: expansion and activation of resident tissue fibroblasts, transition of epithelial cells into mesenchymal cells (epithelial-to-mesenchymal transition [EMT]), and tissue migration of bone marrow-derived circulating fibrocytes [8]. Recently, endothelial-to-mesenchymal transition (EndoMT), a newly recognized type of cellular transdifferentiation, has emerged as another possible source of tissue myofibroblasts [9]. EndoMT is a complex biological process in which endothelial cells lose their specific markers and acquire a mesenchymal or myofibroblastic phenotype and express mesenchymal cell products such as alpha smooth muscle actin (α-SMA) and collagen type I (Col I) [8, 9]. Despite evidence suggesting that EndoMT is involved in not only pathological [10–12] but also physiological conditions [13], the underlying molecular mechanisms involved in this process are largely unknown. Similarly to EMT, EndoMT can be induced by TGF-β [14]. Of note, the same molecule is considered, together with ET-1, to be a pivotal player in the developmental of SSc fibrosis [15]. It is well documented that the activation of TGF-β intracellular transcription factors is responsible of the production of other fibrotic molecules, such as ET-1 [16]. Moreover, TGF-β-induced ET-1 release has been associated with the fibrotic response of skin and lung SSc fibroblasts [17]. In the present study, we established an in vitro model of EndoMT made up of fibroblast and microvascular endothelial cell (MVEC) cocultures. Considering the synergistic action of ET-1 and TGF-β to induce EndoMT, we investigated the potential of ET-1 dual receptor antagonists bosentan (BOS) and macitentan (MAC) to antagonize EndoMT in our in vitro coculture model.

## Methods

### Patient enrollment, skin biopsy, and cell isolation

#### Patients

We enrolled ten women (age 55.7 ± 12.3 years, disease duration 8.3 ± 1.8 years) affected by limited SSc (lSSc) in accordance with the description of LeRoy and colleagues [18] and who fulfilled the 2013 American College of Rheumatology/European League Against Rheumatism diagnostic criteria for SSc [19]. Table 1 shows the major demographic and clinical characteristics of these patients. We performed skin biopsies by using a 3-mm punch on affected skin graded as 2 according to modified Rodnan skin score (mRSS) [20]. Unaffected areas of midforearm skin from the same patients with lSSc were also taken. The unaffected skin of patients with lSSc was defined by clinical palpation (graded as 0 according to mRSS) and by histological examination that excluded SSc-related lesions.

**Table 1:** Clinical parameters of patients with limited systemic sclerosis

Patient	Age (years)	Sex	Disease duration (years)	Digital ulcers	PAH	mRSS/score at affected skin biopsy	ANA-ENA
1	64	F	8	no	no	10/2	Anticentromere-CenpB
2	35	F	9	no	no	09/2	Anticentromere-CenpB
3	72	F	11	yes	yes	13/2	Anticentromere-CenpB
4	49	F	7	no	no	10/2	Anticentromere-CenpB
5	37	F	5	no	no	10/2	Anticentromere-CenpB
6	52	F	10	no	no	11/2	Anticentromere-CenpB
7	59	F	8	yes	yes	13/2	Anticentromere-CenpB
8	66	F	9	no	no	09/2	Anticentromere-CenpB
9	58	F	6	no	no	10/2	Anticentromere-CenpB
10	65	F	10	no	no	10/2	Anticentromere-CenpB
Mean (SD)	55.7 (12.3)		8.3 (1.8)				

*Abbreviations*: *ANA* Antinuclear antibodies, *ENA* Extractable nuclear antigens, *CenpB* Centromere protein B, *mRSS* Modified Rodnan skin score, *PAH* Pulmonary arterial hypertension

Patients discontinued corticosteroids, oral vasodilators, intravenous prostanoids, and other disease-modifying drugs at least 1 month before skin biopsies

#### Fibroblast isolation and cultures

Fibroblasts were isolated from skin specimens by enzymatic digestion. Briefly, explants were de-epidermized using a dispase solution (dispase activity 14 U/ml; Sigma-Aldrich, St. Louis, MO, USA) for 2 h at 37 °C and then were dissolved into a collagenase III solution (2.4 U/ml; Sigma-Aldrich) for 30 minutes. Fibroblasts obtained were passaged twice and cultured at a density of 1 × 10^6^ cells per flask in DMEM (Sigma-Aldrich) supplemented with penicillin (100 U/ml; Sigma-Aldrich), streptomycin (100 μg/ml; Sigma-Aldrich), amphotericin B (0.25 μg/ml; Sigma-Aldrich), glutamine (2 mM; Sigma-Aldrich), and 10 % FBS (Sigma-Aldrich), followed by incubation at 37 °C in an atmosphere of 5 % CO_2_ and 95 % air until confluence (1 week) in 75-cm^2^ flasks (BD Costar, Cambridge, MA, USA). Viability was estimated by trypan blue staining (Sigma-Aldrich). Fibroblasts were used at third passage (P3) for cocultures.

#### MVEC isolation and cultures

Biopsy samples were washed with PBS (Sigma-Aldrich) and placed into a 50-ml tube (BD Costar) containing 15 ml of trypsin (Sigma-Aldrich) for digestion at 37 °C for 45 minutes. Cells were cultured in EGM-MV microvascular endothelial cell growth medium (Lonza, Walkersville, MD, USA) at 37 °C in an atmosphere of 5 % CO_2_ and 95 % air. Before reaching confluence, the heterogeneous pool of cells was exposed to a cluster of differentiation 31 (CD31)-positive selection by using the DynaBeads magnetic CD31 MicroBeads cell-sorting system (Invitrogen, Carlsbad, CA, USA) following the manufacturer’s instructions. After removing CD31-negative cells, only cells positive for CD31 were collected. MVECs obtained were used at P3 for mono- and cocultures.

### EndoMT coculture model

In order to set up an in vitro model of EndoMT, fibroblasts isolated from affected skin biopsies and MVECs isolated from unaffected skin areas were cocultured in Transwell® 12-well plate inserts (0.4-μm pore size; Corning, Corning, NY, USA). Fibroblasts and MVECs were seeded in cocultures in a 1:3 ratio: fibroblasts in transwell inserts and MVECs in the bottom of the wells. The following cocultures of affected fibroblasts with affected MVECs (positive control) and unaffected fibroblasts with unaffected MVECs (negative control) were also performed. The fibroblast layer of the EndoMT model was incubated with 100 nM ET-1 and 5 ng/ml TGF-β for 48 h to permanently maintain the myofibroblast phenotype in culture. The MVEC layer of the EndoMT model was left undisturbed for 48 h or preincubated with 10 μM BOS or 1 μM MAC [6] 1 h before treating the fibroblast layer with ET-1 and TGF-β.

### Tube formation ability of MVECs

Tube formation ability was evaluated using a Matrigel assay. Matrigel (BD Biosciences, San Jose, CA, USA) was used at 8.6 mg/ml in a 1:1 dilution with EGM2-MV (Lonza), without any supplement. MVECs were labeled, before coculture in Matrigel, with the red fluorescent dye PKH26 (Sigma-Aldrich) following the manufacturer’s instructions. After 48 h of mono- or coculture with fibroblasts, MVECs tube total length in each well, calculated as cells per millimeter, were calculated by using a Zeiss AxioPlan2 fluorescence microscope equipped with AxioVision 4.6 for Windows software (Carl Zeiss Vision GmbH, Hallbergmoos, Germany). The number of branching points was calculated using MetaMorph® software for Windows (Molecular Devices, Sunnyvale, CA, USA). The number of tubes formed was quantitated by an observer blinded to the experimental groups.

### Western blot analysis

Collected MVECs were centrifuged at 1500 rpm for 10 minutes at room temperature. Pellets were then suspended in PBS buffer and centrifuged at 3000 rpm for 20 minutes at 4 °C. The obtained pellets were then suspended in radioimmunoprecipitation assay buffer (Sigma-Aldrich) containing protease inhibitor cocktail (Sigma-Aldrich) for 40 minutes in ice and then centrifuged at 15000 rpm for 30 minutes at 4 °C. After centrifugation, supernatants were collected, and protein concentration was calculated according to Bradford assay [21]. About 20 μg of cell lysate proteins for each lane were resolved in a 12 % SDS-PAGE buffer according to Laemmli [22]. The bands were transferred from the gels to polyvinyl difluoride membranes using the iBlot™ Dry Blotting System (Invitrogen). The endothelial markers chosen for MVECs were rabbit polyclonal anti-CD31 (Abcam, Cambridge, UK), rabbit polyclonal anti-vascular endothelial growth factor A (anti-VEGF-A) (Abcam), and rabbit polyclonal anti-VEGF-A165b (Abcam). The mesenchymal markers chosen for MVECs were rabbit polyclonal anti-α-SMA (Abcam), rabbit polyclonal anti-Col-I (Abcam), and rabbit polyclonal anti-TGF-β (Abcam). Incubation was carried out using iBlot® Western Detection Kit (Invitrogen). The load control protein used was beta actin (Abcam). The bound primary antibodies were detected using antirabbit immunoglobulin G alkaline phosphate conjugate (Invitrogen) and visualized using a ChemiDoc™ XRS 170-870 molecular imager (Bio-Rad Laboratories, Hercules, CA, USA) and quantified by using Quantity One software (Bio-Rad Laboratories).

### RNA isolation and quantitative real-time polymerase chain reaction

MVECs were collected in TRIzol reagent (Sigma-Aldrich). Total RNA was extracted following the manufacturer’s instructions. The total RNA content of the samples was quantified by measuring the absorbance at 260 nm using an Ultrospec 2000 spectrophotometer (Amersham Pharmacia Biotech, Piscataway, NJ, USA). The RNA was then reverse-transcribed using a random hexamer MultiScribe enzyme (Applied Biosystems, Foster City, CA, USA). Quantitative real-time polymerase chain reactions (qRT-PCR) were run in the StepOne Real-Time PCR System (Applied Biosystems) using TaqMan chemistry (Invitrogen). Two microliters of complementary DNA in a final volume of 20 μl were amplified using the 20× Assays-on-Demand gene expression assay mix (Applied Biosystems). Specific primers were designed on the basis of the reported sequences (National Center for Biotechnology Information PrimerBank): α-SMA: 5′-CGGTGCTGTCTCTCTATGCC-3′ (forward) and 5′-CGCTCAGTCAGGATCTTCA-3′ (reverse); Col I: 5′-AGGGCCAAGACGAAGACAGT-3′ (forward) and 5′-AGATCACGTCATCGCACAACA-3′ (reverse); TGF-β: 5′-CTAATGGTGGAAACCCACAACG-3′ (forward) and 5′-TATCGCCAGGAATTGTTGCTG-3′ (reverse); CD31: 5′-AACAGTGTTGACATGAAGAGCC-3′ (forward) and 5′-TGTAAAACAGCACGTCATCCTT-3′ (reverse); VEGF-A: 5′-AGGGCAGAATCATCACGAAGT-3′ (forward) and 5′-GCTGCGCTGATAGACATCCA-3′ (reverse); VEGF-A165b: 5′-GAGCAAGACAAGAAAATCCC-3′ (forward) and 5′-GTGAGAGATCTGCAAGTACG-3′ (reverse). TaqMan probes, specific primers and ribosomal 18S, selected as a housekeeping gene, were purchased from Applied Biosystems. Messenger RNA (mRNA) levels were normalized to those of 18S.

### Statistical analysis

Prism 6.0® for Windows software (GraphPad Software, La Jolla, CA, USA) was used for statistical analysis. Data regarding Western blot analyses are expressed as mean ± SD of three technical replicates evaluated by analysis of variance (ANOVA) and Tukey’s multiple-comparisons test. Significance was set at *p* < 0.05. Data related to the number of branching points are expressed as mean ± SD, while those related to tube formation ability and to qRT-PCR are expressed as median (range) of six biological replicates. Because the data followed a nonparametric distribution, the Mann-Whitney *U* test was thought to be appropriate for the analyses. Significance was set at *p* < 0.05.

## Results

### Tube formation ability of MVECs

**Fig. 1:**
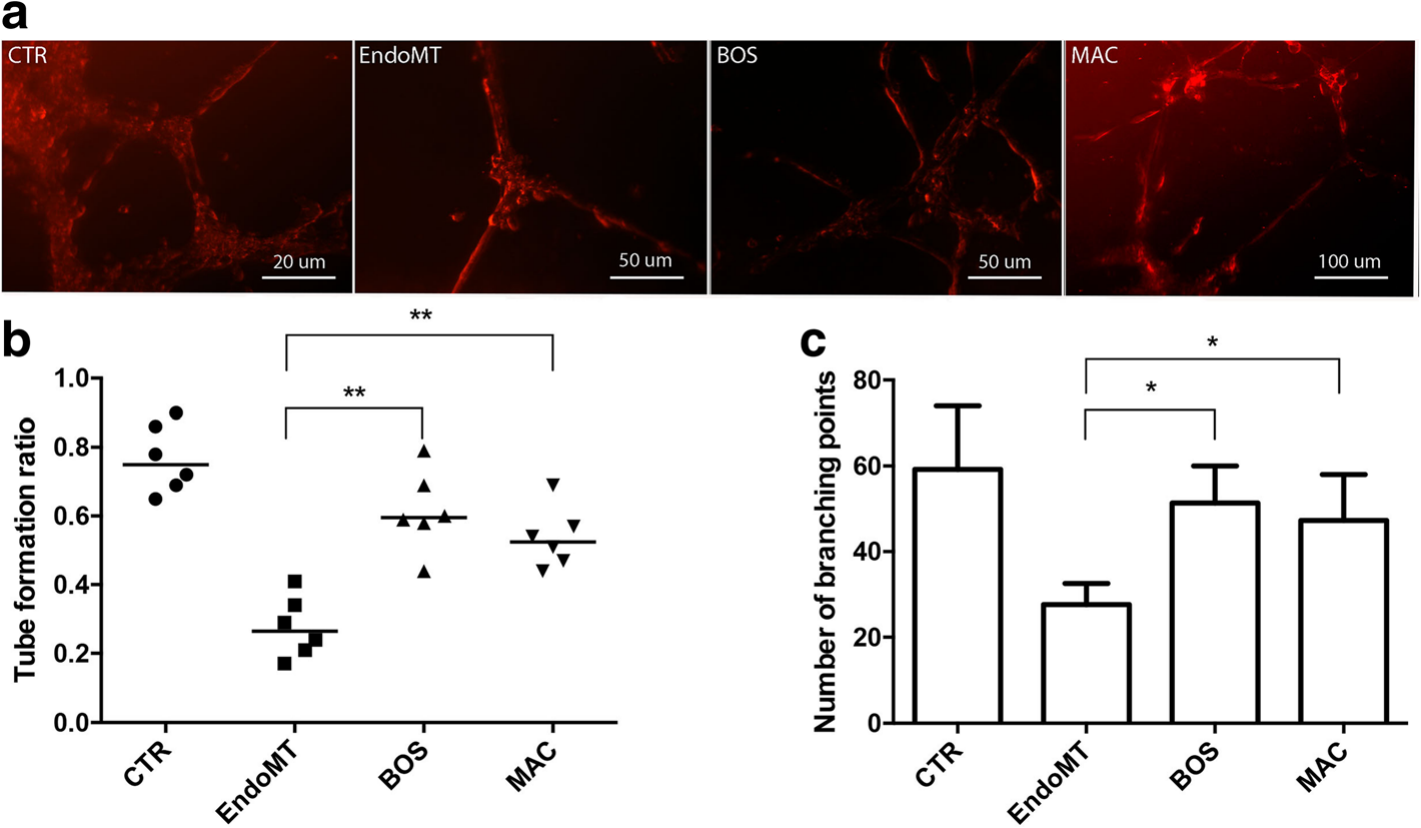
**a** Tubular structure formation of microvascular endothelial cells (MVECs) in Matrigel after 48 h of culture alone (before endothelial-to-mesenchymal transition [EndoMT]) or cocultures with fibroblasts. The fluorescent *red* images show that MVECs (post-EndoMT) have a decreased tube formation ability with respect to those treated with bosentan (BOS) and macitentan (MAC), which show a well-organized tubelike network. **b** The tube formation ability was measured as cells per millimeter and is expressed as the ratio of total tube length in each culture condition to the length in the culture of untreated (CTR) MVECs. Data are expressed as median (range) of six biological replicates (*p < 0.05, **p < 0.01). **c** Number of branching points expressed as mean ± SD of six biological replicates (*p < 0.05)

In our in vitro EndoMT model, MVECs cocultured with affected fibroblasts (stimulated with ET-1 and TGF-β) showed a significant impairment in tube formation, probably due to the persistent stimulation of the myofibroblast phenotype (Fig. 1a). After 48 h of coculture, MVECs statistically reduced their ability to form tubular structures in untreated samples. In fact, as shown in Fig. 1b, the analysis of tube formation ratio evidenced significant improvement in tube formation when MVECs were preincubated with BOS (*p* < 0.01) and MAC (*p* < 0.01). These data were confirmed by analysis of the number of branching points (Fig. 1c) that were statistically increased when MVECs were incubated with BOS (*p* < 0.05) and MAC (*p* < 0.05).

### Effect of BOS and MAC on endothelial marker expression in MVECs

**Fig. 2:**
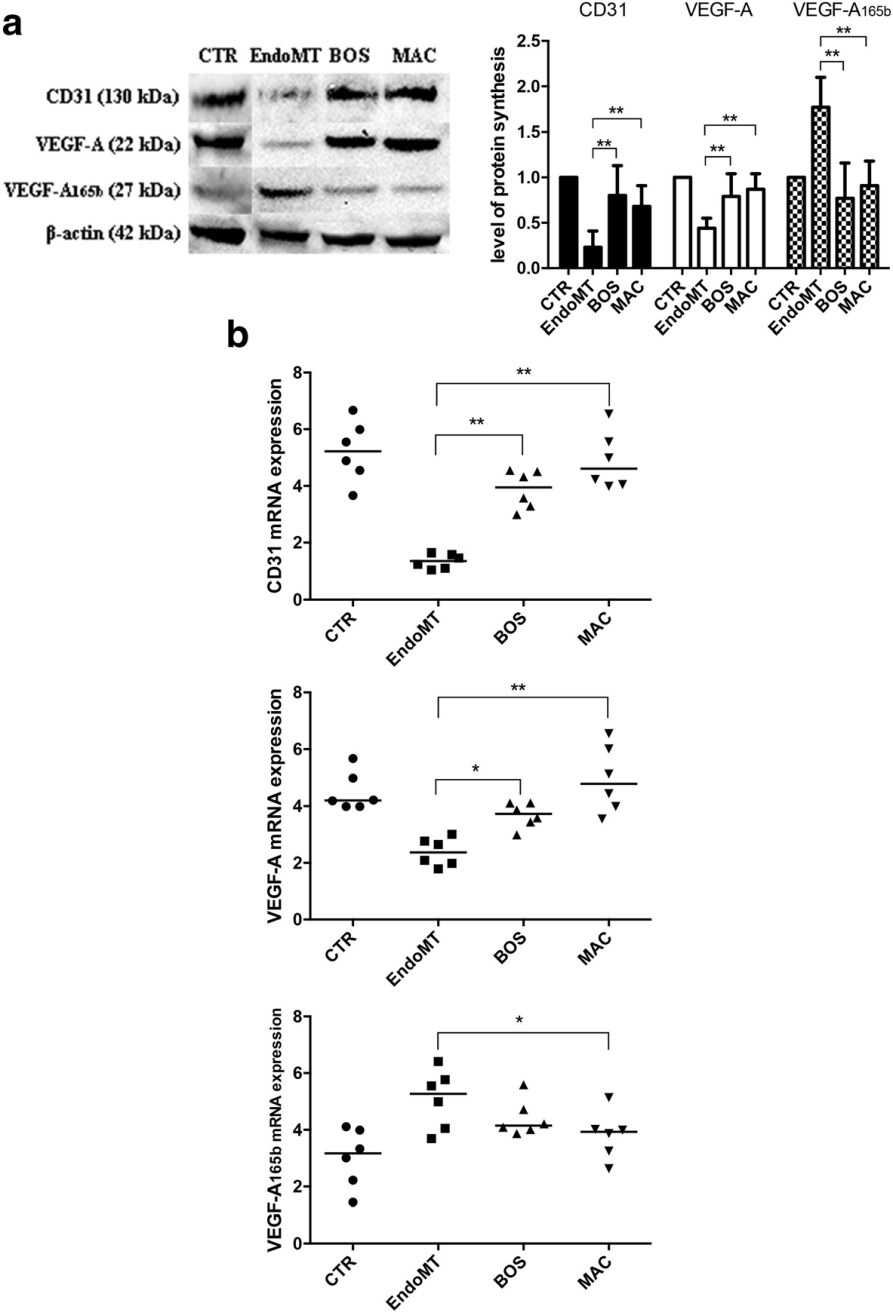
**a** Western blot analyses (*left*) and the relative densitometric values (*right*) of cluster of differentiation 31 (CD31), vascular endothelial growth factor (VEGF)-A, and VEGF-A165b. Densitometric data are representative of three technical triplicates and are expressed as mean ± SD. The values of protein synthesis obtained for each treatment (bosentan [BOS] and macitentan [MAC]) were normalized to that of the untreated cells (CTR) taken as unit value by definition. Analysis of variance and Tukey’s multiple-comparisons test were performed for each group (*p < 0.05, **p < 0.01). **b** Quantitative real-time polymerase chain reaction analyses for CD31 (*top*), VEGF-A (*middle*), and VEGF-A165b (*bottom*) confirm the results observed in Western blot analysis. The results are expressed as median (range) of six biological replicates (*p < 0.05, **p < 0.01). *mRNA* Messenger RNA; *EndoMT* Endothelial-to-mesenchymal transition

As shown in Fig. 2, both BOS and MAC are able to antagonize the EndoMT process after 48 h. In particular, 1 h of preincubation with 10 μM BOS led to both protein (*p* < 0.01) and mRNA (*p* < 0.01) increases of CD31, and also of both protein (*p* < 0.01) and mRNA (*p* < 0.05) increases of VEGF-A. At the same time, the antiangiogenic VEGF-A165b protein (*p* < 0.01) but not mRNA levels statistically decreased. Conversely, 1 h of preincubation with 1 μM MAC led to both protein (*p* < 0.01) and mRNA (*p* < 0.01) increases of CD31, and also of both protein (*p* < 0.01) and mRNA (*p* < 0.01) increases of VEGF-A. VEGF-A165b protein (*p* < 0.01) and mRNA (*p* < 0.05) levels statistically decreased.

### Effect of BOS and MAC on mesenchymal marker expression in MVECs

**Fig. 3:**
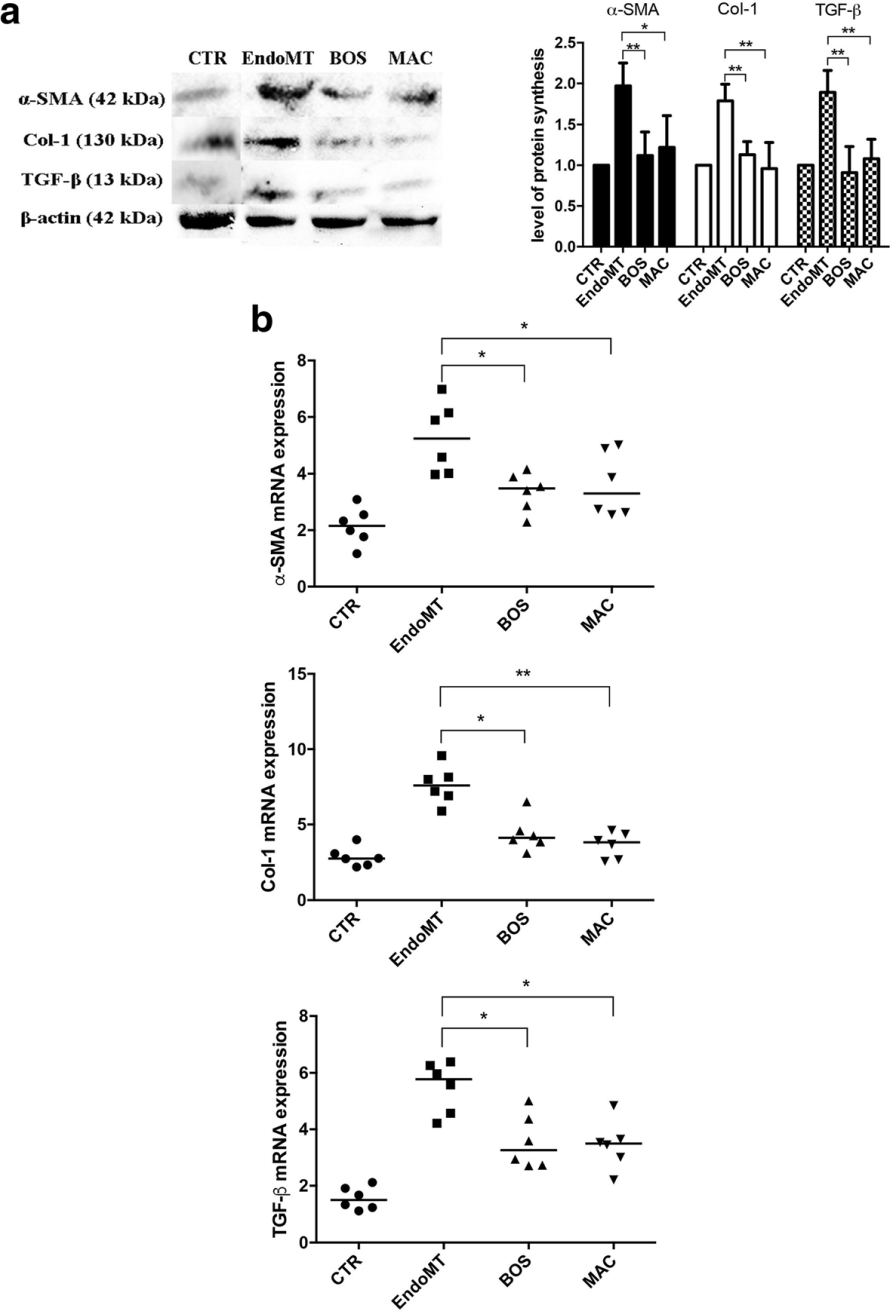
**a** Western blot analyses (*left*) and the relative densitometric values (*right*) of alpha smooth muscle actin (α-SMA), collagen type I (Col I) and transforming growth factor beta (TGF-β). Densitometric data are representative of three technical replicates and are expressed as mean ± SD. The values of protein synthesis obtained for each treatment (bosentan [BOS] and macitentan [MAC]) were normalized to that of the untreated cells (CTR), taken as unit value by definition. Analysis of variance and Tukey’s multiple-comparisons test were performed in each group (*p < 0.05, **p < 0.01). **b** Quantitative real-time polymerase chain reaction analyses for α-SMA (*top*), Col I (*middle*), and TGF-β (*bottom*) confirm results obtained by Western blot analysis. The results are expressed as median (range) of six biological replicates (*p < 0.05, **p < 0.01). *mRNA* Messenger RNA; *EndoMT* Endothelial-to-mesenchymal transition

Figure 3 shows that BOS and MAC are able to antagonize the EndoMT through the expression of typical mesenchymal markers in MVECs. In particular, BOS is responsible for both protein (*p* < 0.01) and mRNA (*p* < 0.05) decreases of α-SMA, protein (*p* < 0.01) and mRNA (*p* < 0.05) decreases of Col I, and protein (*p* < 0.01) and mRNA (*p* < 0.05) decreases of TGF-β. MAC is responsible for both protein (*p* < 0.05) and mRNA (*p* < 0.05) decreases of α-SMA, protein (*p* < 0.01) and mRNA (*p* < 0.01) decreases of Col-1, and protein (*p* < 0.05) and mRNA (*p* < 0.01) decreases of TGF-β.

## Discussion

The in vitro experimental model of EndoMT described in the present study provides evidence that the persistence of the myofibroblast phenotype in SSc and its mediators (ET-1 and TGF-β) are responsible for the transdifferentiation of MVECs toward the mesenchymal phenotype [23, 24]. As reported in the literature, the onset of SSc is represented mainly by vascular damage [4, 25], but this EndoMT in vitro model could explain the persistence of fibroblast-vascular crosstalk as a profibrotic loop-promoting condition in SSc. In fact, the recruitment of myofibroblasts in affected tissues, associated with their elevated biosynthetic functions, may be considered as pivotal determinants of the extent and progression rate of the fibrosis in SSc [26]. Using a tridimensional Matrigel model, we showed the reduction of MVECs’ ability to form tubular structures when cocultured with affected fibroblasts incubated with ET-1 and TGF-β. In the same condition, it was possible to observe a switch from proangiogenic to antiangiogenic VEGF isoforms, as recently described in the literature [27, 28]. The overexpression of VEGF165b, an inhibitory splice variant of VEGF, leads to insufficient angiogenesis in patients with SSc [29]. Not only the VEGF family but also CD31, the specific endothelial marker, seems to be downregulated after 48 h of coculture during the EndoMT, a condition recently described in the literature [30, 31]. MVECs lose specific endothelial markers and increase mesenchymal profibrotic markers, such as α-SMA, Col I, and TGF-β [32]. This evidence allowed us to hypothesize that the above-mentioned profibrotic switch may be considered a normal response of MVECs in any physiological (wound healing) [33] and pathological (SSc) [34] conditions characterized by overexpression of ET-1 and TGF-β. In this setting, considering the synergistic action of ET-1 and TGF-β in triggering EndoMT [35], our study strengthens the fundamental therapeutic utility that blocking the ET-1 system, by using dual endothelin-1 receptor antagonists (ERA), could represent an important therapeutic strategy [36]. Our in vitro model demonstrates that both BOS and MAC are effective in inhibiting the ET-1/TGF-β-mediated EndoMT, supporting the hypothesis that ET-1 may represent the ultimate mediator of TGF-β actions [37, 38]. It is very important to highlight that not only MAC but also BOS is effective in EndoMT antagonism in vitro. Therefore, it could be hypothesized that the obtained results depend more on ET-1 receptor (ET_A_ and ET_B_) blocking than on the physiochemical and pharmacokinetic properties of the ERA used, even though a functional in vitro assay demonstrated that the potency of MAC is tenfold higher than that of the parent compound BOS [39]. Translating these preclinical findings into clinical applications, we hypothesize that the therapeutic strategy of antagonizing EndoMT could contribute to counteract endothelial dysfunction in pulmonary arterial hypertension [40, 41], a frequent condition in patients with SSc [42], and to counteract the steps leading to SSc digital ulcerations [43]. In fact, the remodeling effects of ERA on microvascular damage progression in patients with SSc who have undergone long-term treatment have already been demonstrated by nailfold videocapillaroscopy and laser Doppler analysis of fingertip blood flow [44–46].

## Conclusions

The present study provides further in vitro evidence of the use of ERA (BOS and MAC) in inhibiting the EndoMT process, supporting the clinical efficacy of these drugs in SSc therapy and their usefulness for interfering with progressive fibrosis.

## Abbreviations

ANA: Antinuclear antibodies
ANOVA: Analysis of variance
BOS: Bosentan
CD31: Cluster of differentiation 31
CenpB: Centromere protein B
Col I: Collagen type I
EGM-MV: Microvascular endothelial cell growth medium
EMT: Epithelial-to-mesenchymal transition
ENA: Extractable nuclear antigen
EndoMT: Endothelial-to-mesenchymal transition
ERA: Dual endothelin 1 receptor antagonists
ET-1: Endothelin 1
lSSc: Limited systemic sclerosis
MAC: Macitentan
mRNA: Messenger RNA
mRSS: Modified Rodnan skin score
MVEC: Microvascular endothelial cell
P3: Third passage
PAH: Pulmonary arterial hypertension
qRT-PCR: Quantitative real-time polymerase chain reaction
SSc: Systemic sclerosis
TGF-β: Transforming growth factor beta
VEGF: Vascular endothelial growth factor
α-SMA: Alpha-smooth muscle actin
